# Local tetanus begins with a VAMP cleavage-associated neuromuscular junction paralysis around the site of tetanus neurotoxin release

**DOI:** 10.1016/j.ajpath.2024.05.009

**Published:** 2024-06-15

**Authors:** Federico Fabris, Aram Megighian, Ornella Rossetto, Morena Simonato, Giampietro Schiavo, Marco Pirazzini, Cesare Montecucco

**Affiliations:** 1 Department of Biomedical Sciences, University of Padova, Via Ugo Bassi 58/B, 35131 Padova, Italy; 2 Padova Neuroscience Center, University of Padova, Via Giuseppe Orus 2, 35131 Padova, Italy; 3 Center of Myology CIR-Myo, University of Padova, Via Ugo Bassi 58/B, 35131 Padova, Italy; 4 Institute of Neuroscience, National Research Council, Via Ugo Bassi 58/B, 35131 Padova, Italy; 5 Department of Neuromuscular Diseases and UCL Queen Square Motor Neuron Disease Centre, UCL Queen Square Institute of Neurology, London WC1 3BG, United Kingdom; 6 UK Dementia Research Institute at University College London, London WC1 3BG, United Kingdom

**Keywords:** local tetanus, tetanus neurotoxin, neuroparalysis, neuromuscular junction, spinal cord

## Abstract

Local tetanus develops when limited amounts of tetanus neurotoxin (TeNT) are released by *Clostridium tetani* generated from spores inside a necrotic wound. Within days, a spastic paralysis restricted to the muscles of the affected anatomical area develops. This paralysis follows the retrograde transport of TeNT inside the axons of spinal cord motoneurons and its uptake by inhibitory interneurons with cleavage of VAMP, a synaptic vesicle protein required for neurotransmitter release. Consequently, incontrollable excitation of motoneurons causes contractures of innervated muscles and to local spastic paralysis.

Here, the initial events occurring close to the site of TeNT release were investigated. A peripheral flaccid paralysis was found to occur, before or overlapping, the spastic one. At variance from the confined TeNT proteolytic activity at the periphery, central VAMP cleavage can be detected within inhibitory interneurons controlling motor neuron efferents innervating muscle groups distant from the site of TeNT release. These results indicate that TeNT does have a peripheral activity in tetanus and explains why the spastic paralysis observed in local tetanus, although confined to single limbs, generally affects multiple muscles. The initial TeNT neuroparalytic activity can be detected by measuring the compound muscle action potential providing a very early diagnosis and therapy thus preventing the ensuing life-threatening generalized tetanus.

## Introduction

Tetanus is a life-threatening disease which has afflicted vertebrates, including humans, for millennia ^1^. Toxigenic strains of *Clostridium tetani* have been recently found associated with mummies discovered in different parts of the world ^2^. Tetanus begins with the infection of necrotic wounds by *C. tetani* spores which germinate under anaerobiosis generating bacteria that multiply and release a 150 kDa protein exotoxin, named tetanus neurotoxin (TeNT), which is the sole responsible for all tetanus symptoms ^3–7^. The most frequent form of tetanus is a generalized spastic paralysis that affects all skeletal muscles with painful contractures beginning from the head (trismus, or lockjaw), then descending to the neck (opisthotonos) and thorax, eventually causing cardiorespiratory failure, sometimes involving autonomic dysfunctions ^3–6^. Maternal and neonatal tetanus are two high fatality forms present in low-income countries where tetanus vaccination is incomplete ^8, 9^, and please see https://www.who.int/news-room/fact-sheets/detail/immunization-coverage (last access may 8, 2023). Diagnosis of these forms of tetanus is mainly clinical and rather unmistakable given the evidence of symptoms like trismus, opisthotonos, dysphagia, respiratory embarrassment and muscle spasms. A rarer form is local tetanus, characterized by a spasticity limited to the muscles adjacent to the necrotic wound, which may initially be confused with other diseases in humans and pets ^10–16^. Cephalic tetanus is another uncommon form of tetanus. It derives from a head wound and frequently displays cerebral palsy before spasticity of head and neck muscles develop, leading to an initial mis-diagnosis which renders this form of tetanus particularly insidious because it may rapidly progress to a generalized tetanus ^17, 18^.

Tetanus has almost disappeared from most of the world thanks to a very effective and low-cost vaccination with tetanus toxoid, which elicits the formation of immune memory cells producing toxin-specific TeNT-neutralizing antibodies that can protect from disease for dozens of years. However, with time, the level of immunization decreases and a boost with a vaccine dose is necessary to extend the immunization ^1, 4, 19, 20^. Regrettably, tetanus continues to be present in some African and Asian developing countries due to incomplete vaccination. However, an efficacious passive immunization of skin-wounded patients is available and it is a common practice in Emergency Units; it is based on immunoglobulins isolated from antisera obtained from hyperimmune humans or horses ^1, 3–5^.

Together with botulinum neurotoxins, TeNT forms the growing family of the structurally-related clostridial neurotoxins which are the most poisonous substances known to mankind with lethal doses in the low ng/kg range in mice ^21^. These neurotoxins consist of a metalloprotease light chain (L, 50 kDa) linked to a heavy chain (H, 100 kDa) by a peptide belt and a single interchain disulphide bond whose integrity is essential for neurotoxicity ^22–27^. The two polypeptide chains are folded into three domains, each of 50 kDa: the globular N-terminal domain is the catalytic L chain endowed with a Zn-endopeptidase activity, the elongated alpha helical intermediate H_N_ domain is involved in the membrane translocation of the L chain into the cytosol and the C-terminal H_C_ domain that mediates TeNT binding to the presynaptic membrane ^23–25^. The L chain of TeNT cleaves at a single site the vesicle associated membrane protein (VAMP), also termed synaptobrevin, at a single site thus preventing the release of neurotransmitter at nerve terminals ^28–31^.

The accepted view on tetanus pathogenesis is that TeNT binds to peripheral nerve terminals and is taken up inside the lumen of signalling endosomes, which are retrogradely transported inside the axon to the perikaryon of motoneurons, from where the toxin is released in the spinal cord fluids. TeNT then binds spinal GABAergic and glycinergic inhibitory interneurons and it is taken up inside the acid lumen of endocytic compartments ^32–43^. Low pH triggers the translocation of the L chain from the lumen to the cytosolic face of the organelle membrane ^44, 45^. The reduction of the interchain disulfide bridge by the Thioredoxin-Thioredoxin reductase system frees the metalloprotease activity with VAMP cleavage and consequent blockade of neurotransmitter release ^27, 46^. The ensuing loss of inhibitory control causes hyperexcitability and excitation of motor neurons which in turn elicits contractures of the innervated muscles. This process first affects the muscles of the region where the wound containing *C. tetani* is localized causing a local tetanus. If enough toxin is produced, it diffuses in the body and binds to the presynaptic unmyelinated area of additional motor, sensory and autonomic axon terminals. The retrograde transport of TeNT to the spinal cord affects several skeletal muscles, including respiratory muscles, causing a generalized tetanus ^7, 35^.

It was recently reported that in cephalic tetanus, cerebral palsy is caused by the blockade of neuromuscular junctions (NMJ) around the wound site caused by entry of TeNT within presynaptic motor axon terminals with local VAMP cleavage. This peripheral activity transiently precedes and can overshadow the central action of TeNT, thus permitting a rapid dissemination of TeNT in the spinal cord before tetanus symptoms become apparent ^18^.

Here, the hypothesis that a spatially restricted TeNT-induced flaccid paralysis is also present in local tetanus was tested and the dissemination of TeNT activity within the spinal cord following its retrograde axonal transport was examined. TeNT was found to cleave VAMP within the motor axon terminal of NMJs located around the toxin release site, whilst leaving untouched those terminals located further away. At variance, VAMP cleavage attains a larger distribution in central synapses.

The present results indicate that an initial flaccid paralysis is a general feature of local tetanus pathophysiology and that, upon retrograde axonal transport, TeNT disseminates throughout the spinal cord affecting different motor efferents. These findings explain why the spastic paralysis of local tetanus, although confined to single limbs, generally affects multiple muscles around the site of spore entry.

## Materials and Methods

### Materials

TeNT was purified from a culture of *C. tetani* (strain Harvard), aliquoted and maintained at - 80 °C as previously described ^47^. TeNT was thawed, gently stirred, left o/n at room temperature and diluted to the desired concentration with phosphate buffered saline (PBS) solution containing 0.2% gelatin (cat. G2500, Sigma) prior to use. The atoxic binding fragment of tetanus neurotoxin (H_C_T, residues 875-1315) fused to a cysteine-rich tag and a human influenza haemagglutinin epitope was expressed in *E. coli*, purified as a glutathione-S-transferase fusion protein and labelled with AlexaFluor555 maleimide (Life Technologies, A-20346), as previously described ^48^. Anti cl-VAMP (1:1000) was generated in rabbit and affinity purified as described before ^49, 50^. Anti VAChT (cat. 139105, 1:500) and anti VAMP-2 (cat. 104211, 1:500) were purchased from Synaptic System (Göttingen, Germany), Alexa-647-conjugated α-bungarotoxin was from Life Technologies (cat. B35451, 1:200, goat anti-rabbit conjugated to Alexa-555 (cat. A21428, 1:200), goat anti-guinea pig conjugated to Alexa 488 (cat. A11073, 1:200) and goat anti-mouse conjugated to Alexa 647 (cat. A21235, 1:200) were purchased from Thermo Fisher Scientific.

### End Plate Potential (EPP) recordings

Evoked EPP were performed as previously described ^51^. Briefly, CD1 mice weighting around 25–30 gr were anesthetized with isoflurane gas, the soleus muscle was surgically exposed and injected with TeNT (0.5 ng/Kg contained in 2 µl) or vehicle using a homemade micro injector. Mice were then left to recover under infrared lights and then monitored to evaluate the progression of local tetanus. As soon as tetanus paralysis begun (2-3 days), mice were anesthetised with a cocktail of xylazine (48 mg/Kg) and Zoletil (16 mg/Kg) via i.p. injection and euthanized. Soleus muscles were quickly dissected out and the tendons were pinned on the bottom of a Sylgard-coated Petri dish. Recordings were performed in oxygenated (95% O_2_, 5% CO_2_) Krebs-Ringer solution via intracellular microelectrodes (glass, 1.5 mm outer diameter, 1.0 mm inner diameter, 15-20 MΩ tip resistance; GB150TF, Science Products GmbH Germany), filled with one part of a 3M KCl, and 2 parts of a 2M CH_3_COOK solution. Neuromuscular transmission was recorded in current-clamp mode, and resting membrane potential was adjusted with current injection to -70 mV. EPPs were elicited by supramaximal nerve stimulation at 0.5 Hz using a suction microelectrode (GB150TF, Science Products GmbH Germany) connected to a S88 stimulator (Grass, USA). Muscle fibres contraction during intracellular recordings was blocked by adding 1 μM μ-Conotoxin GIIIB (Alomone Lab, Israel) to avoid cell membrane damage due mechanical movements. Intracellular signals were processed with an intracellular amplifier (BA-01X, NPI, Germany). Using a digital A/C interface (NI PCI-6221, National Instruments, USA), amplified signals were converted to digital format and fed to a computer for data recording using an appropriate software (WinEDR, Strathclyde University; pClamp 10.3, Axon, USA). Stored data were analysed off-line using the software pClamp 10.3 (Axon, USA). At the end of the experiment, soleus, gastrocnemius (medialis and lateralis) and tibialis anterior muscles were processed for immunofluorescence.

### Electromyographic recordings

CD1 mice weighting 25–30 g were subcutaneously inoculated with TeNT (0.5 ng/Kg contained in 5 µl) or vehicle in the hind paw pad after isoflurane gas anaesthesia. After 48 hours, mice were anesthetised with a cocktail of xylazine (48 mg/Kg) and Zoletil (16 mg/ Kg) i.p. and the sciatic nerve was exposed at the trochanter level, without damaging the musculature. A small piece of parafilm was put under the nerve kept hydrated with a drop of saline (0.9% NaCl in deionized water). Compound Muscle Action Potentials (CMAPs) were then evoked stimulating the exposed nerve with a pair of needle electrodes placed on the nerve using a mechanical micromanipulator (MM33, FST, Germany). Stimulating electrodes were connected via a Stimulus Isolation Unit (SIU5, Grass, USA) to a stimulator (S88, Grass, USA). Supramaximal stimuli (25V, 0.5 ms duration) were delivered at 0.5 Hz in a capacitive coupling mode. Recording and reference electrodes (Grass) were inserted in the flexor digitorum brevis muscle (FDB) halfway in the hind paw pad and under the skin of the ankle, respectively. Recorded signals were filtered between 0.1 Hz and 100 kHz, amplified using an extracellular amplifier (P6 Grass, USA), digitized using a digital A/C interface (National Instruments, USA) and then fed to a computer for recording (WinEDR, Strathclyde University; pClamp 10.3, Axon, USA). Stored data were analysed offline using pClamp 10.3 software (Axon, USA). At the end of the experiment, FDB muscles were collected, fixed in 4% paraformaldehyde in PBS and processed for immunofluorescence.

### H_C_T-555 diffusion assay

CD1 mice weighting around 25–30 gr were anesthetized with isoflurane gas and the soleus muscle was surgically exposed. H_**C**_T-555 (1 µg in 2 µl) was injected using a homemade micro injector consisting of a Hamilton syringe (full volume 25 µl) connected to a micrometer with a mechanical counter (Mitutoyo, Dusseldorf DE, usable range 0 – 25 mm, resolution 0.01 mm). The syringe tip is inserted into a 20 cm long rubber tube filled with paraffin oil equipped with a 28G needle for subcutaneous injections. The syringe was loaded with 5 µl of toxin solution, the needle delicately inserted in the soleus muscle for the injection. The injection pace is 200 nL per micrometer turn. Mice were left to wake up under infra-red light. After 4 hours the low hind limb muscles, including the soleus, the medial and lateral gastrocnemii and the tibialis anterior were collected and immediately fixed with 4% PFA in PBS. Muscles were then separated in bundles of 15-20 fibers and stained with α-bungarotoxin conjugated with Alexa-647 in PBS for 1 h at room temperature. After extensive washes with PBS, fibers were mounted on coverslips with Dako mounting medium (cat F4680, Merk Life Science, Italy). Images were collected with a Zeiss confocal microscope equipped with a N-Achroplan 20x/0.45 Ph2 M27-Air Objective, a Plan-Apochromat 40x/0.95 Korr M27-Oil immersion Objective, or a Plan-Apochromat 63x/1.4 DIC M27-Oil immersion Objective. Laser excitation line, power intensity and emission range were chosen according to each fluorophore in different samples to minimize bleed-through.

### Immunofluorescence

Immediately after electrophysiology, muscles were fixed with 4% paraformaldehyde in PBS for 30 min at room temperature and then quenched in PBS containing 0.24% of NH_4_Cl for 20 min. Muscles were then dissected in bundles of five-to-ten fibers, permeabilized in blocking solution (15% goat serum, 2% BSA, 0.25% gelatin, 0.20% glycine, 0.5% Triton X-100 in PBS) and incubated for 72 h with the indicated primary antibodies diluted in blocking solution at 4˚C. Muscles were then washed three times in PBS and incubated with secondary antibodies diluted in PBS + 0.5% Triton X-100 supplemented with Alexa-647-conjugated α-bungarotoxin for 1 h at room temperature. After extensive washes with PBS, muscle fibers were mounted on coverslips with Dako mounting medium (cat F4680, Merk Life Science, Italy).

For spinal cord staining, mice were intracardially perfused with 50 ml of PBS followed by 30 ml of 4% paraformaldehyde in PBS, the spinal cord collected, post fixed with 4% paraformaldehyde, 15% sucrose in PBS, and then stored at 4 °C in PBS containing 30% sucrose. Spinal cords were cut in 20 µm thick slices using a cryostat, permeabilized in blocking solution and incubated for 24 hours with primary antibodies diluted in blocking solution. Slices were then washed three times in PBS and incubated with the appropriate secondary antibodies diluted in PBS + 0.5% Triton X-100 for 1h at room temperature. After extensive washes with PBS, slices were mounted on coverslips using Dako mounting medium.

Images were collected with a Zeiss confocal microscope equipped with a N-Achroplan 20x/0.45 Ph2 M27-Air Objective, a Plan-Apochromat 40x/0.95 Korr M27-Oil immersion Objective, or a Plan-Apochromat 63x/1.4 DIC M27-Oil immersion Objective. Laser excitation line, power intensity and emission range were chosen according to each fluorophore in different samples to minimize bleed-through.

### Statistical analysis

Sample sizes were determined by analysis based on data collected by our laboratory in published studies. A minimum number of 4 mice per group was used in each experiment (immunofluorescence staining, electrophysiological analysis) with blinding conduct of experiments through compartmentalization. GraphPad Prism software was used for statistical analyses and to pull out track peaks. Statistical significance was evaluated using unpaired Student’s t-test or by one-way analysis of variance (ANOVA). Data were considered statistically different when ** p < 0.01, *** p < 0.001.

### Ethical Statement

All experimental procedures involving animals and their care comply with the ARRIVE guidelines. Procedures were approved by the ethical committee and by the animal welfare coordinator of the OPBA from the University of Padua. All procedures are specified in the projects approved by the Italian Ministry of Health, Ufficio VI (authorization numbers: 359/2015 PR; 81/2017 PR; 521/2018 PR; 439/2019 PR) and were conducted in accordance with national laws and policies (D.L. n. 26, March 14, 2014), following the guidelines established by the European Community Council Directive (2010/63/EU) for the care and use of animals for scientific purposes. Animals were handled by specialized personnel under the control of inspectors from the Veterinary Service of the Local Sanitary Service (ASL 16-Padua), who are the local officers of the Ministry of Health. Mice were purchased from Charles River Laboratories and maintained under 12 h light/dark cycle in a controlled environment fed with regular chow and with water provided ad libitum. All procedures involving mice were performed under general anesthesia and analgesia.

## Results

### TeNT rapidly paralyses the injected muscles by cleaving VAMP and blocking acetylcholine release at the neuromuscular junction

**Figure 1:**
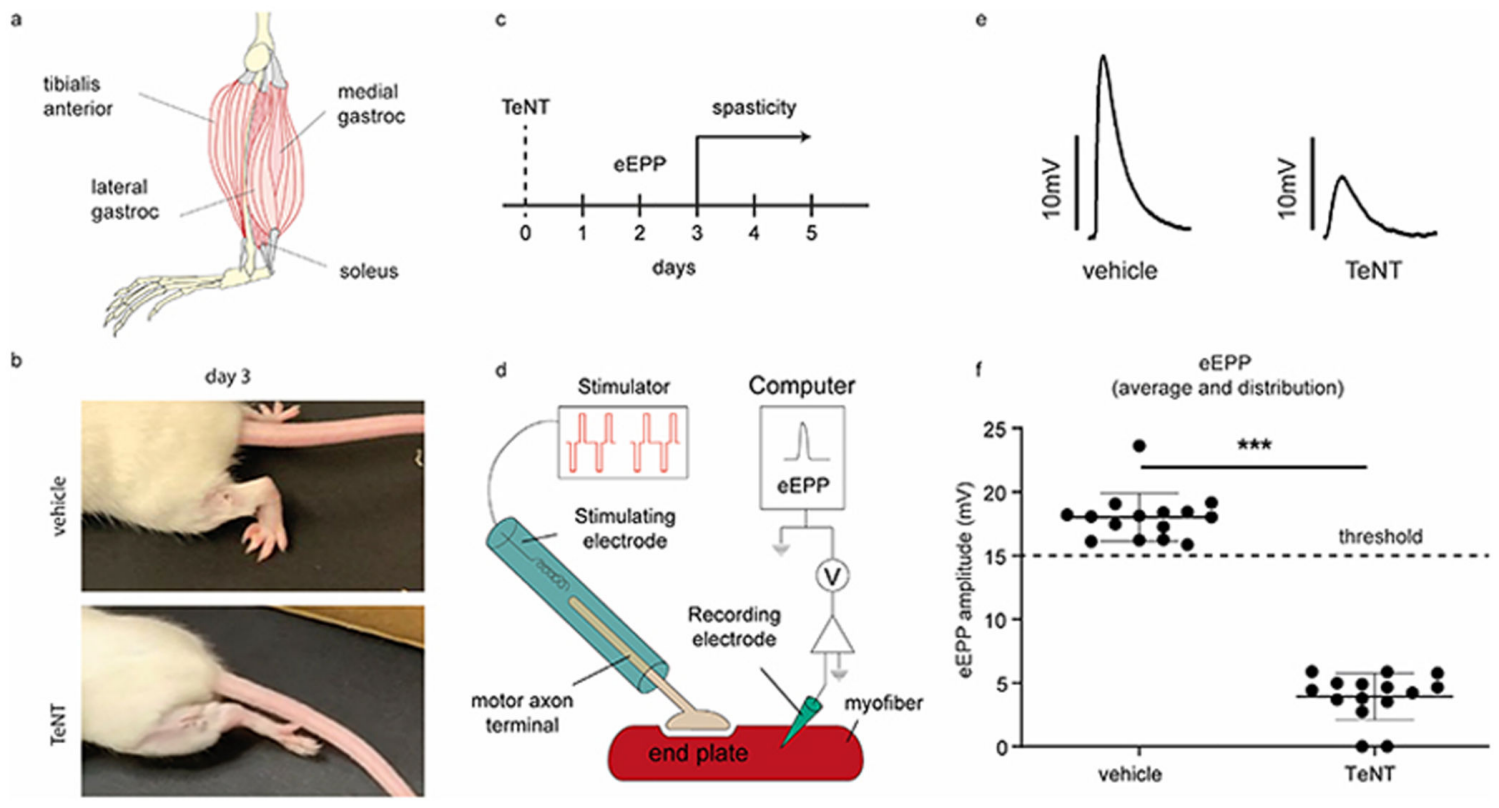
Soleus-specific injection of TeNT leads to local tetanus in the hindlimb preceded by a localized flaccid paralysis of the muscle. **(A)** Schematic representation of the mouse hindlimb musculature showing the muscle-specific injection of TeNT (0.5 ng/kg in 2 µl) into the soleus muscle (dark red) using a glass capillary syringe. (**B)** Representative video frame image of the local spastic paralysis affecting the mouse hindlimb 72 hours after the soleus-specific injection of TeNT (upper panel). A representative video frame of a vehicle-injected animal is shown in the lower panel. **(C)** Timeline showing the time course of eEPP electrophysiology measurements 2 days after the soleus-specific injection of TeNT, i.e., 1 day before hindlimb spastic paralysis is attained. **(D)** Scheme showing the experimental setup to measure neurotransmitter release at the neuromuscular junction via eEPP assessing neurotransmission with single myofiber resolution. The soleus muscle is isolated with its intact nerve and placed in a petri chamber with a physiologic solution. The nerve is electrically stimulated to evoke the endplate potential then measured with a recording electrode inserted into individual fibers. **(E)** Representative traces and **(F)** amplitude distribution of eEPP recorded in single soleus myofibers injected with either the vehicle (left panel) or 0.5 ng/kg TeNT (right panel). Each point represents the mean amplitude of at least 20 EPPs evoked in a single fiber. Data are from three animals per group with at least five myofibers analysed each, and represent the distribution of values, group mean, and standard deviations. Statistical analysis was performed using t-test with Welch’s correction *** p <0.001.

To test the possibility that in local tetanus TeNT cleaves VAMP within local motor axon terminals, thus causing a localized flaccid paralysis before spasticity develops, a dose of 0.5 ng/Kg of highly purified TeNT was injected within the mouse soleus muscle (Figure 1A). In preliminary experiments this dose of TeNT was found to reproducibly cause local tetanus characterized by a significant, yet spatially restricted, spastic paralysis of the injected limb (Figure 1B). When limb spasticity begun to be noticeable (2/3 days after, Figure 1C), the soleus muscles were collected and the evoked End Plate Potential (EPP) was measured. EPP provides an accurate assessment of neuromuscular transmission with single synapse resolution (Figure 1D) ^51^. The EPP amplitude largely decreased (Figure 1E), indicating a strong impairment of NMJ transmission.

**Figure 2:**
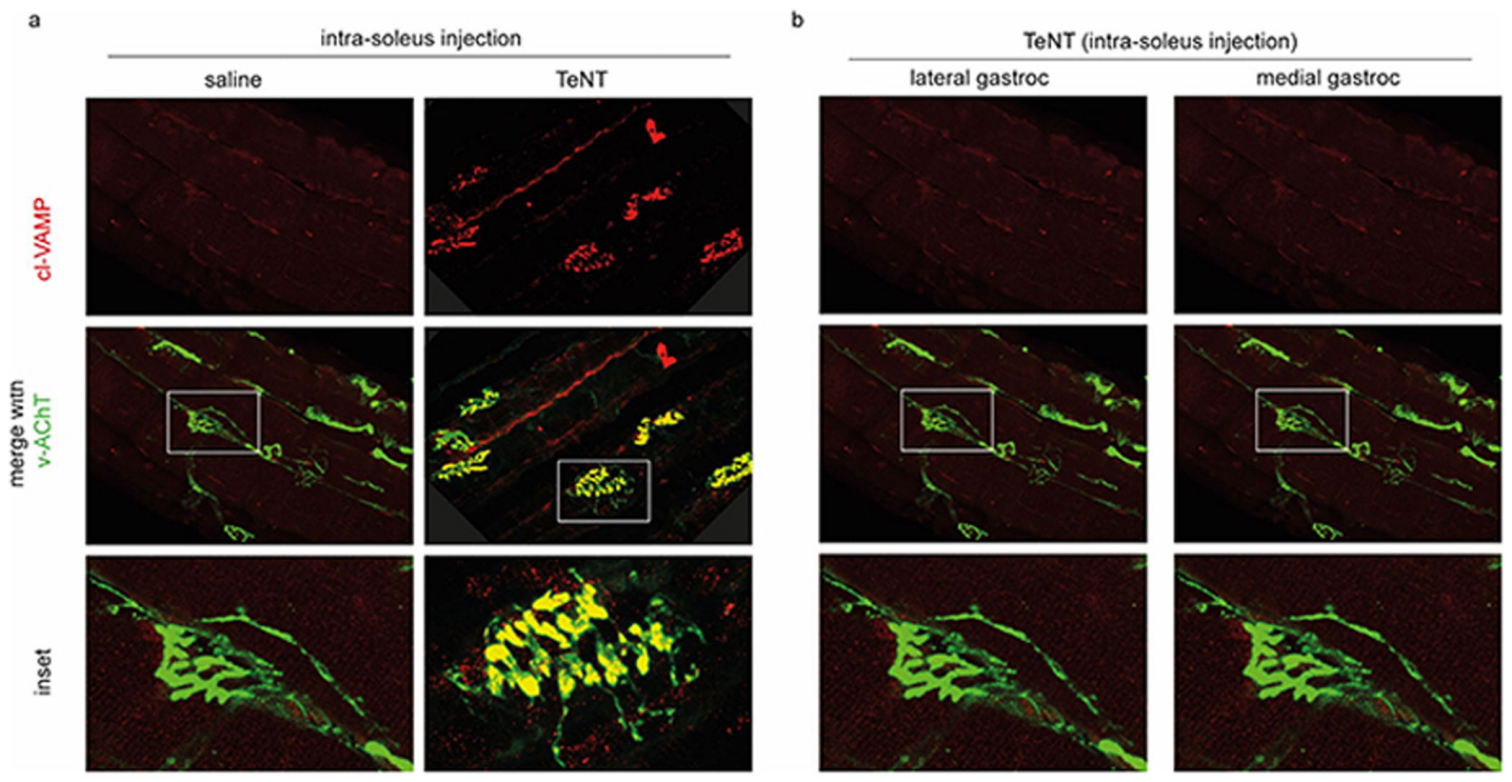
TeNT displays a peripheral activity following soleus-specific injections. Representative images of motor axon terminals at the NMJ from soleus, medial and lateral gastrocnemius and tibialis anterior muscles upon soleus-specific injection of vehicle or TeNT (0.5 ng/kg). The red staining is for cl-VAMP, which specifically detects the proteolytic activity of TeNT whilst the green staining is from α-bungarotoxin-Alexa 488 to detect AChR. No VAMP cleavage signal is detected in the saline-injected muscle neither in muscles surrounding the injected soleus, indicating that the peripheral activity of TeNT is limited to the muscle directly injected. The white boxes indicate the area considered for the insets. Images are representative of at least three independent experiments. Scale bars are 50 µm or 25 µm for the insets.

After electrophysiology was performed, the soleus muscles were PFA-fixed and teased in small bundles of myofibers for immunostaining with an antibody specific for the TeNT-cleaved form of VAMP (cl-VAMP). This approach provides a highly amplified, *in situ* imaging of the TeNT metalloprotease activity ^49, 50^. Figure 2 shows that soleus motor axon terminals display a clear staining of cl-VAMP, facing the post synaptic membrane stained with alpha-bungarotoxin. Cleavage of VAMP prevents the assembly of the apparatus that mediates the fusion of synaptic vesicle with the presynaptic membrane with the consequent inhibition of acetylcholine release and blockade of the NMJ function ^28,30,31^. At the same time, no cleavage of VAMP was detected in adjacent muscles, including the lateral and medial gastrocnemius that are physically in contact with the injected soleus muscle, indicating that TeNT peripheral activity was restricted to the anatomical site of toxin injection where the concentration was sufficiently high to intoxicate nerve terminals.

### Muscular paralysis induced by TeNT measured by the compound muscle action potential (CMAP)

**Figure 3:**
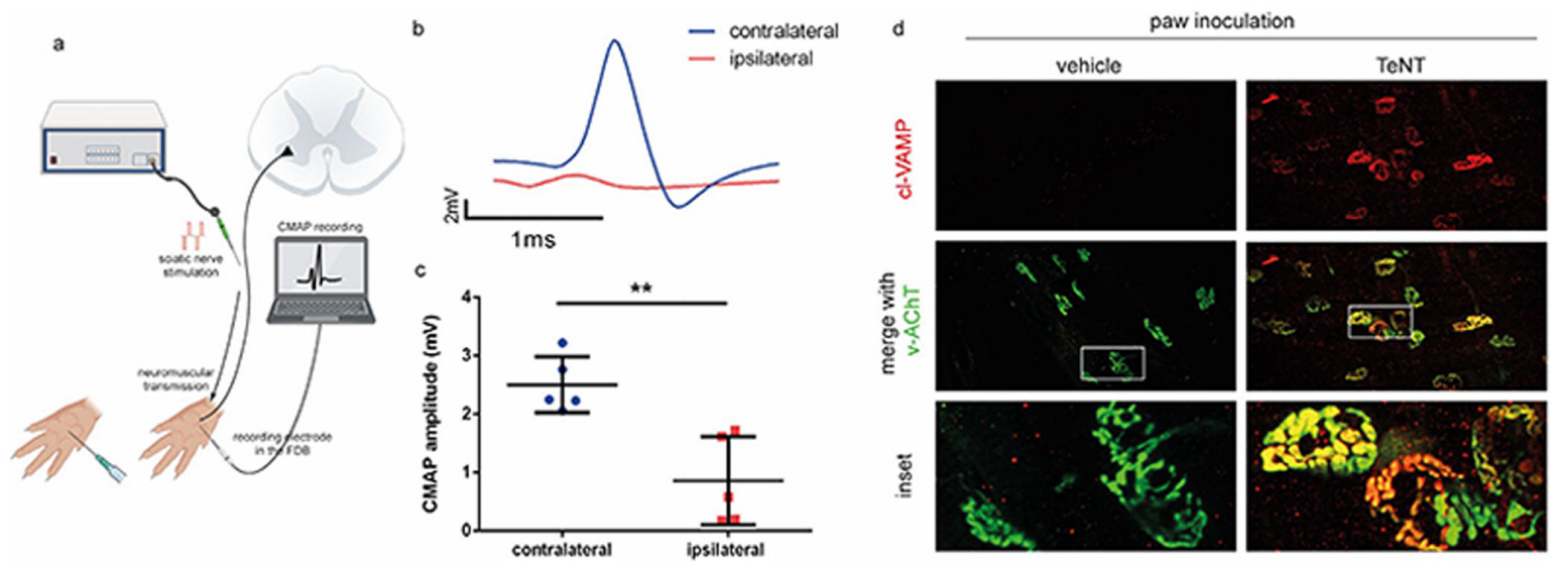
The CMAP response of the FDB muscle is strongly reduced after TeNT inoculation in the hind paw. **(A)** Schematic representation of TeNT (0.5 ng/kg in 5 µl) inoculation in the mouse hind paw followed 2 days after by FDB electromyography upon sciatic nerve electrical stimulation in living animals. **(B)** Representative single CMAP traces elicited by supramaximal sciatic nerve stimulation (0.5 Hz) and recorded in the FDB of the injected limb (red trace) and in the contralateral limb injected with the vehicle (blue). **(C)** Quantification of CMAP amplitude in vehicle injected and TeNT-inoculated hind paws. Each point represents the mean value obtained from 10 CMAP evoked in one out of five individual animals. Statistical analysis was performed by t-test with Welch correction, ** p-value: 0.01. (**D)** Representative images of motor axon terminals at the NMJ from the FDB muscles upon inoculation of either a vehicle or TeNT (0.5 ng/kg in 5 µl) stained for cl-VAMP (red) and the synaptic vesicle marker VAChT (green). The white boxes indicate the area considered for the insets. Scale bars are 50 µm or 25 µm for the insets.

The soleus-specific injection represents a useful system to quantitatively assess the TeNT paralytic action at the NMJ via measurement of EPP. However, measurement of EPP and cl-VAMP detection at the NMJ cannot be performed in humans. To overcome this limitation, TeNT was injected in the mouse paw thus mimicking foot wounding, and neurotransmission was measured in the flexor digitorum brevis (FDB) muscle by detecting the CMAP (Figure 3A). This test is routinely performed in human patients, affected by neuromuscular disorders or neuropathies, to assess the activity of peripheral nerves in patients. Consistent with the EPP decrease, TeNT caused a marked reduction in the CMAP of the FDB upon sciatic nerve stimulation, which provides a clear demonstration of peripheral neuromuscular paralysis (Figure 3B, C). The paralysis was demonstrated to be directly caused by TeNT by the detection of extensive VAMP cleavage at local NMJs (Figure 3D). Of note, all these pathological changes were detected 2 days after toxin inoculation when limb spasticity and/or muscle contractures begun to be noticeable. This suggests that a peripheral local TeNT-induced VAMP proteolysis develops at the NMJ before the central effects of TeNT, causing muscle contractures, are detectable.

### TeNT diffusion from the site of muscle injection is very limited

**Figure 4:**
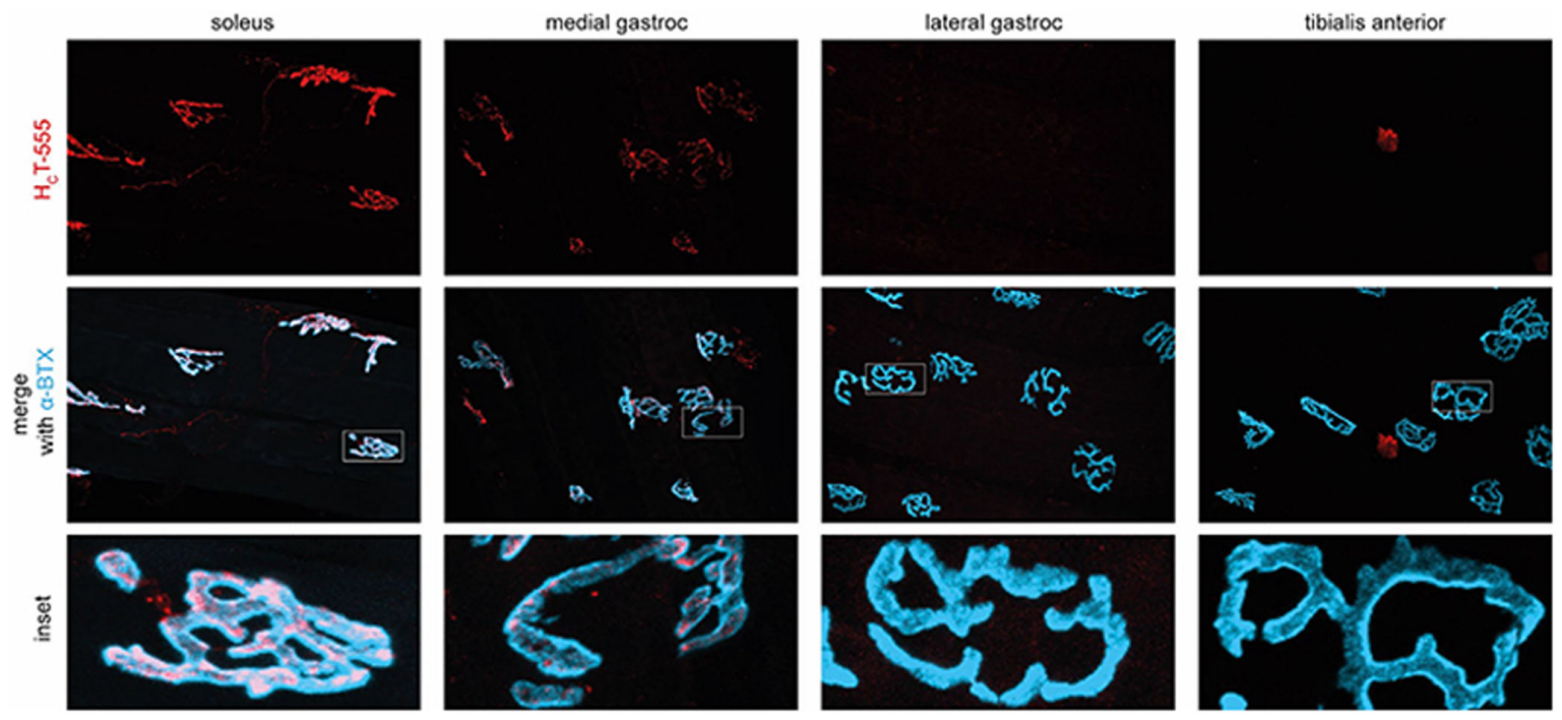
TeNT has minimal peripheral diffusion upon soleus-specific injection. **(A)** Schematic representation of the mouse hindlimb musculature and of the muscle-specific injection of H_c_T-555 (1 µg in 2 µl) into the soleus (dark red) using a glass capillary syringe. (**B)** Time course of the experiment showing the dissection for NMJ imaging of hindlimb muscles (soleus, medial gastrocnemius, lateral gastrocnemius, and tibialis anterior) 4 hours after H_c_T-555 injection. **(C)** Representative images of motor axon terminals at the NMJ from soleus, medial and lateral gastrocnemius and tibialis anterior muscles upon soleus-specific injection of H_c_T-555 (1 µg in 2 µl). The red staining is for HcT-555 whilst the green staining is from α-bungarotoxin-Alexa 488 to detect AChR. The white boxes indicate the area considered for the insets. Scale bars are 50 µm or 25 µm for the insets

Both soleus- and FDB-inoculated muscles eventually led to local limb spastic paralysis of tetanus. It is important to note that, notwithstanding TeNT was injected in a confined area, muscle contracture and spastic paralysis affected several hindlimb muscles. This situation can derive either from TeNT dissemination within the spinal cord after retroaxonal transport or from the diffusion of TeNT in the rest of the body via lymphatic and blood circulation followed by retroaxonal transport upon toxin entry of several motor axon terminals, or both. Although the latter possibility is less likely as the toxin presumably dilutes several folds in these processes becoming incapable of binding to peripheral presynaptic terminals, the peripheral diffusion was tested after injecting within the soleus the H_C_ binding domain of TeNT labelled with a fluorescent dye emitting at 555 nm (H_C_-TeNT-555). Whilst the efficiency of internalisation and trafficking of the fragments of TeNT might be different from that of the entire toxin^52^, the ability of H_C_-TeNT and its fluorescent derivatives to enter and undergo fast retrograde transport in neurons *in vitro* and *in vivo* has been widely documented ^41, 53–55^, as well as its property of accumulating at the neuromuscular junction and in the spinal cord upon injection in a variety of muscles ^54, 56^. Figure 4 shows that the probe injected in the soleus muscle stains local NMJs but not those in adjacent muscles, except for a faint staining of the NMJs of the medial, but not lateral, gastrocnemius, a muscle adjacent to soleus. The situation is different when *C. tetani* in the necrotic wound continues to produce TeNT that may diffuse at distance with transformation of a local tetanus in a general tetanus.

### TeNT causes a diffuse VAMP cleavage within the spinal cord after the initial peripheral proteolysis

**Figure 5:**
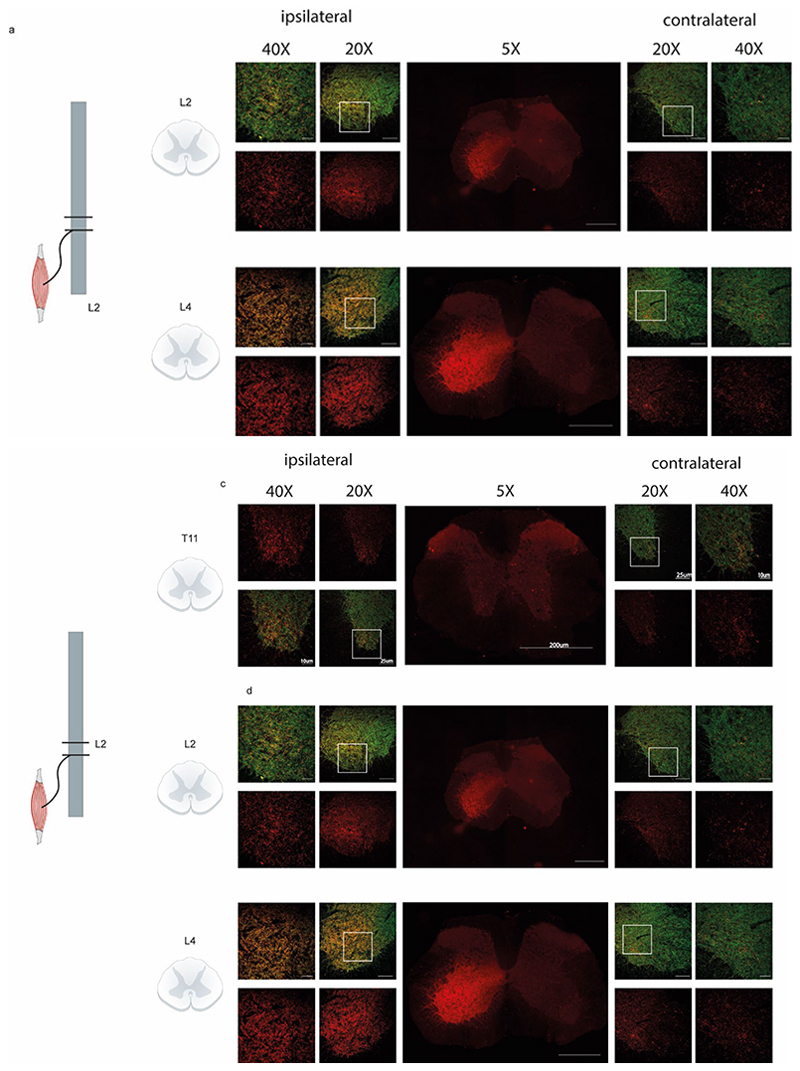
TeNT enters the spinal cord and spreads its proteolysis of VAMP throughout motor nuclei controlling several hindlimb muscles. **(A)** Schematic representation of L1-L6 motor nuclei and of their axons innervating the indicated hindlimb muscles. As shown in the scheme, each motor nucleus does extend across multiple vertebrae. **(B)** Top panel shows a representative low-magnification tile-scan image of cl-VAMP (red) staining in the ipsilateral (ipsi) and contralateral (contra) ventral horns of a L4 transverse cryosection taken from a mouse injected with TeNT into the left soleus. Scale bar is 200 µm. The middle panel shows a schematic representation of the motor nuclei present in L4 as reported in ^60^. The red area corresponds to the posterior crural nucleus containing the motor efferents innervating the soleus. The bottom panel displays a representative tile-scan of the L4 transverse section in pseudocolors to show the radial distribution of the cl-VAMP signal around the central white region corresponding to the maximum signal, as indicated by the colour scale. Scale bar, 200 µm. **(E)**. The top left panel shows a representative image of cl-VAMP staining (red) in the L4 ventral horn. The numbered white boxes indicate the area of the different motor nuclei displayed in the other panels in an enlarged view with the corresponding numbers. Scale bars are 25 µm for the top left panel and 10 µm for the remaining panels. **(F)** Representative images of cl-VAMP (red) colocalizing with VGAT (green), a marker of inhibitory axon terminals. Scale bars are 5 µm (left panel) or 2.5 µm (middle and right panels). **(E-H)** These panels are the same as A-D but are taken from the transverse L2 cryosection.

Immunofluorescence imaging of cleaved VAMP was performed in two transverse sections of the spinal cord (Figure 5): L4 which includes perikaryons of motoneurons innervating muscles of the lower and upper limb (Figure 5A), and L2 which does not include neurons connected to the soleus where the toxin was injected ^60^. An extensive staining of cl-VAMP is clearly detectable in the ventral horn of L4 (Figure 5B). The signal appears very intense at the level of the crural motor nuclei, which includes the motoneurons innervating the soleus those which transported TeNT in the lumbar segment of the spinal cord. However, staining radially extended to nearby motor nuclei innervating the muscles of the entire hind limb (Figure 5C, D). At higher magnification, cl-VAMP was detected within the motor nuclei of lateral and posterior crural muscles, including the soleus, gastrocnemius, tibialis and plantaris muscles, of the adductor muscles, of the iliopsoas muscles of the quadriceps and of the gluteal muscles (Figure 5E). Co-staining with VGAT reveals that in all these regions cl-VAMP is predominantly present within GABAergic and glycinergic terminals (Figure 5F). Interestingly, a strong signal of cl-VAMP is present also in the ventral horn of L2 despite the fact that the soleus, where the toxin was injected, does not have motor efferents at this level (Figure 5G-J), indicating vertical diffusion of the enzymatically active TeNT within the ipsilateral side of the spinal cord. cl-VAMP was found in several motor nuclei at the level of inhibitory axon terminals also in this segment of the spinal cord (Figure 5K-L), which is consistent with the spreading of contractures and spasticity to multiple muscles of the injected leg, a characteristic of local tetanus.

In addition, at both L4 and L2 levels, diffusion of TeNT activity in the contralateral side was detected although the signal is very limited compared to the ipsilateral side and not always present within GABAergic and glycinergic axon terminal markers (Figure 1S). Similar findings were obtained in cats using ^125^I-TeNT, a toxin radiolabeled with a procedure that largely inactivates the toxin and that can form toxin fragment by radiolysis ^35^. The present data extend this observation of TeNT spinal diffusion made in the poorly TeNT sensitive cats using a very high quantity of toxin, to a highly TeNT sensitive species such as *Mus musculus* and using a limited amount of fully active toxin.

## Discussion

The main results presented here provide an experimental demonstration that in local tetanus a blockade of the NMJs located around the site of TeNT injection/release develops following the cleavage of VAMP inside their motor axon terminals. As this biochemical lesion prevents the fusion of synaptic vesicles and granules ^30, 31^, the NMJ is no longer functional. Most likely, such a local paralysis has gone so far unnoticed because of its small entity and possibly because it is then obscured by the intervening contracture and spastic paralysis of the muscles around the site of toxin release. This novel knowledge has been gathered by using a recently developed antibody that recognizes VAMP specifically cleaved at a single peptide bond by TeNT ^49^. This method is highly sensitive in detecting the proteolytic activity of TeNT *in situ*, i.e., inside nerve terminals where TeNT has entered and acted enzymatically. In parallel, the paralysis of NMJ was monitored at a single muscle fiber level by measuring the evoked end plate potential and in the whole muscle by measuring the compound muscle action potential with results in agreement with a previous study ^61^. The distribution of TeNT after i.m. injection has been previously studied using rats and goldfish (see for example ^62–64^) and a local paralytic action of TeNT was reported. However, it should be noted that these animal species are resistant to tetanus and, therefore, very high local concentrations of TeNT had to be used.

The central action of TeNT at the level of spinal cord and brainstem neurons has been well documented before using several experimental approaches (see ^7, 32–35, 37–44^ and references cited therein) and fully explains the cardinal symptoms of tetanus. Parallelly, TeNT was assumed not to act at the peripheral level, unless at exceedingly high concentrations ^62–64^. Here, this generally accepted notion was not supported by the experimental data in a highly tetanus sensitive animal species such as mice. Moreover, using the mouse whisker pad as a model of cephalic tetanus in rodents experimental evidence indicated that TeNT initially causes cerebral palsy via VAMP cleavage within motor neuron terminals of the NMJ innervating facial muscles ^18^.

This situation mirrors the one observed for botulinum neurotoxins (BoNT). These toxins are structurally similar to TeNT, yet they block peripheral nerves causing botulism. Until a few years ago, they were assumed not to act centrally notwithstanding evidence of their central action were available ^65^. This assumption was experimentally proven to be untenable in a series of experiments that visualized the activity of BoNT in central neurons and their retrograde axonal transport in signaling endosomes similar, if not identical, to those transporting TeNT from the NMJ to the spinal cord ^66^.

We believe that TeNT and BoNT action is governed by their local concentration which determines their capacity to bind the neurotoxins receptors present at nerve terminals. Both TeNT and BoNT bind polysialogangliosides as receptors ^67, 68^ and to different protein components ^24, 69–71^. Their dissociation constant (Kd), a measure of their affinity for the nerve terminal, has not been measured *in vivo* but their high toxicity suggests it to be below picomolar. TeNT and BoNTs can be produced or delivered to defined anatomical sites and then diffuse in the body via lymphatic and blood circulation. Their local concentration at the site of production/introduction may be high and therefore one can conceive that: i) TeNT, in addition to those receptors mediating its retroaxonal transport (specific high affinity ganglioside-protein TeNT receptors,) may be capable of binding to the ganglioside-BoNT protein receptors though with a lower affinity, thus permitting the entry of TeNT into peripheral nerve terminals (local action); and *vice versa* ii) BoNTs, in addition to the reported peripheral ganglioside-protein BoNT receptors, may bind to TeNT receptors responsible for retrograde axonal transport. Accordingly, BoNTs, like TeNT, may be transported versus the central nervous system ^66, 72^. In other words, TeNT and BoNT are capable of cross-binding to an extent that it is determined by their local concentration and by the type of nerve terminals involved. A major role in this cross-binding may be played by polysialogangliosides, which were demonstrated long ago to bind both TeNT and BoNT with high affinity ^68, 73–79^. Indeed, this affinity value may be in the same, or close to, concentration value of the toxin at the site of its production or in its very proximity. As the neurotoxins diffuse away into the animal body they dilute substantially. Such a process has not been quantified, but it is bound to lead to a major dilution of the toxin thus rendering receptor cross-binding impossible.

Another important point is related to the distribution of TeNT-cleaved VAMP within the spinal cord. The present findings obtained with a fully active TeNT extends those previously found using ^125^I-radiolabeled TeNT ^36^ and show limited lateral diffusion of the toxin to the contralateral side and a more relevant vertical diffusion above the cell body of the motor neurons innervating the soleus muscle fibers. Other studies performed with electron microscopy autoradiography of ^125^I-radiolabeled TeNT concluded that, after presynaptic uptake, the toxin was carried by retrograde axonal transport to the cell body of neurons. Additional toxin was found in dendrites and in presynaptic terminals on neurons forming synapses with the transporting cell and it was concluded that TeNT undergoes a transynaptic movement to neurons forming synapses with the toxin transporting neurons ^38, 39^. However, these findings do not exclude the possibility that TeNT may also be released from the cell body and dendrites in the intercellular spinal fluids. This latter possibility is supported by an autoradiographic study of the distribution of ^125^I-TeNT in the spinal cord after injection in the gastrocnemius muscle of cat ^36^. The different modes of diffusion are relevant with respect to the therapeutic value of injecting anti-TeNT immunoglobulins intrathecally to intercept TeNT molecules in the spinal cord fluids before they enter neurons and cleave VAMP. At the present stage, it is reasonable to assume that both modes of TeNT diffusion take place within the spinal cord.

In conclusion, the present data provide strong evidence that before or concomitantly to the spastic paralysis of local tetanus, TeNT enters and cleaves VAMP within peripheral motor axon terminals close to the site of toxin release. This activity causes a local neuroparalysis that is detectable via a simple electromyography test such as CMAP. Although extension to human patients is still to be performed, the present data indicate that flaccid paralysis of muscles surrounding a *C. tetani*-infected wound with release of TeNT is a general initial feature of clinical tetanus. This feature may have a diagnostic and clinical relevance that could be assessed by performing a CMAP analysis because it would provide a very early diagnosis of tetanus thus improving the following clinical treatment of the patient thus reducing the risk of the progression versus a life-threatening generalized form of tetanus.

## Data Availability

Data base sharing is not applicable to this article as no new data were created or analyzed in this study.
